# Transkingdom network analysis provides insight into host-microbiome interactions in Atlantic salmon

**DOI:** 10.1016/j.csbj.2021.01.038

**Published:** 2021-01-29

**Authors:** Marius A. Strand, Yang Jin, Simen R. Sandve, Phil B. Pope, Torgeir R. Hvidsten

**Affiliations:** a Faculty of Biosciences, Norwegian University of Life Sciences, 1432 Ås, Norway; b Faculty of Chemistry, Biotechnology and Food Science, Norwegian University of Life Sciences, 1432 Ås, Norway

**Keywords:** Transkingdom network analysis, Host-microbiome interactions

## Abstract

**Background:**

The Atlantic salmon gut constitutes an intriguing system for studying host-microbiota interactions due to the dramatic environmental change salmon experiences during its life cycle. Yet, little is known about the role of interactions in this system and there is a general deficit in computational methods for integrative analysis of omics data from host-microbiota systems.

**Methods:**

We developed a pipeline to integrate host RNAseq data and microbial 16S rRNA amplicon sequencing data using weighted correlation network analysis. Networks are first inferred from each dataset separately, followed by module detections and finally robust identification of interactions via comparisons of representative module profiles. Through the use of module profiles, this network-based dimensionality reduction approach provides a holistic view into the discovery of potential host-microbiota symbionts.

**Results:**

We analyzed host gene expression from the gut epithelial tissue and microbial abundances from the salmon gut in a long-term feeding trial spanning the fresh-/salt-water transition and including two feeds resembling the fatty acid compositions available in salt- and fresh-water environments, respectively. We identified several host modules with significant correlations to both microbiota modules and variables such as feed, growth and sex. Although the strongest associations largely coincided with the fresh-/salt-water transition, there was a second layer of correlations associating smaller host modules to both variables and microbiota modules. Hence, we identify extensive reprogramming of the gut epithelial transcriptome and large scale coordinated changes in gut microbiota composition associated with water type as well as evidence of host-microbiota interactions linked to feed.

## Graphical abstract

**Figure:**
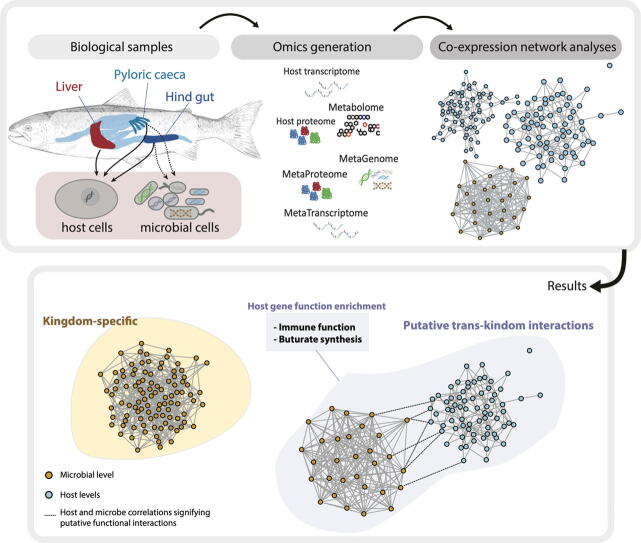

## 1 Introduction

Plants and animals are hosts to a myriad of bacteria, archaea, fungi, protozoa and viruses that make these multicellular organisms their home. One prominent example is the diverse microbial community (the microbiota) residing in the gut of most animals. The formation and preservation of a healthy gut microbiome contributes not only to the extraction of nutrients, but also to normal physiological development of the host including the gastrointestinal tract and the immune system. Indeed, imbalances in the gut microbiota have been associated with obesity, irritable bowel syndrome, asthma, arthritis and even anxiety [39].

The collection of species comprising the host and the microbes living on or inside the host is sometimes referred to as the holobiont, with some evolutionary biologists suggesting that holobionts should be considered single units of selection [43], [7]. Interactions between host and microbiota has been studied in model animals and humans [36], [10], [9], but less is know about such interactions in aquatic environments [11]. To this end, the Atlantic salmon represents an compeling study system due to its intriguing life history. The salmon starts its life in rivers, then migrates to the ocean to mature, and finally returns to the same river to breed. The migration from fresh- to salt-water would kill most fish species, and requires the salmon to undergo substantial changes in the regulation of body chemistry to successfully adapt (smoltification). This transition also represents a barrier to the gut microbiota, which in addition has to adapt to the differences in food between rivers and the ocean. Juvenile fish (parr) in rivers mostly eat invertebrates, which are low in long-chain polyunsaturated fatty acids (LC-PUFAs) such as omega-3, while post-smolt fish in the sea have ample supplies of LC-PUFAs in the form of other fish and krill. Data sets now exist measuring changes in host gene regulation [15] and gut microbial composition [20], [35] as a response to the fresh-/salt-water transition and to different feed types. However, these datasets have so far only been analyzed individually with traditional methods. In this article, we aim to study interactions between the salmon and its gut microbiota by data integration.

Previous studies that have attempted to investigate holobiont interactions by generating large-scale omics data from both the host and the associated microbiota have been limited by a deficit of computational tools and methods [30]. Networks naturally describe interactions, and network-based computational methods have therefore dominated efforts to tease apart such interactions. [19] used networks to identify host gene modules and correlated these with bacterial community variables and individual operational taxonomic units (OTUs) of the microbiome. TransNet (Transkingdom Network, [34] infers unweighted networks of bacterial OTUs and host genes separately. The method relies on differential expression analysis to reduce the number of nodes in the network, followed by network inference, module identification and finally identifies causal host and microbial nodes using high bipartite betweenness centrality in transkingdom networks. Attempts have also been done to infer structural host-microbiota interaction networks by predicting complexes formed between host proteins and microbial components [16], [4]. Here, we develop and apply a network-based dimensionality reduction method for analysing holobiont omics data. The pipeline infers networks for both the host and the microbiota and provides robust predictions of putative Interactions between them. The framework is largely automatic requiring little manual tinkering and is based on weighted networks that do not depend on arbitrary thresholds on correlation [18]. The developed pipeline is then applied to the previously described salmon gut omics datasets.

## 2 Results

To gain insight into host-microbiota interactions in the Atlantic salmon (*Salmo salar*) gut, we performed an integrative analysis of RNA-seq data from the gut epithelial tissue [15] and 16S rRNA amplicon sequencing data from the gut content [20], [35] collected from a long-term feeding trial (Supplementary Fig. 1). The trial spanned fresh- and salt-water life stages and included feeds low (VO – Vegetable Oil) and high (FO – Fish Oil) in long-chain polyunsaturated fatty acids (LC-PUFAs), resembling food availability in rivers and the ocean, respectively. After preprocessing (see Methods), the dataset contained matching measurements of 37,408 host genes and 296 microbiotic OTUs in 147 samples.

### 2.1 A novel method for holobiont interaction analysis

We developed a computational pipeline for integrating host and microbiota omics datasets based on weighted network analysis (Supplemental Fig. 2). We infer networks for each omics dataset separately using the Weighted Gene Co-expression Network Analysis (WGCNA) method [24]. Briefly, networks are soft-thresholded to reach an approximate scale free topology, network modules are detected using the robust weighted Topological Overlap Measure (wTOM) [42] and hierarchical clustering, and the first principle component is used as a representative profile for each module (generally referred to as host/microbiota Module Eigennodes – hME/mME, or, specifically as eigengenes/eigenOTUs). We then assign functional roles at the network module level via function enrichment analysis, while high resolution analysis of the individual modules is enabled through ranking of genes and OTUs (i.e. nodes) by network centrality. Finally, we devised an integrated heatmap analysis to visualize and predict putative transkingdom interactions by correlating module eigennodes between the two networks, and by associating these predicted interactions to host traits and other external variables such as feed and water type.

### 2.2 Fresh-/salt-water transition dominates omics associations

Our analysis of the host gene co-expression network revealed 171 distinct gene modules with low correlation between modules (between host Module Eigengenes – hMEs) and high correlation between genes within the same module (Supplementary Fig. 3). These modules contained 13,525 genes (Supplementary table 1). The microbiota network contained five modules harboring 95 OTUs (Supplementary table 2). Although these modules were distinct, they were clearly clustered into two negatively correlated groups (Supplementary Fig. 4). The relatively large fraction of genes (64%) and OTUs (68%) not included in modules indicates that many genes and bacterial species have largely unique variation in abundance profiles across the individual fish sampled in this study. In an unsupervised framework, such genes/OTUs are assumed to largely make up genes that do not vary (e.g. housekeeping genes) or that display variation not relevant for the processes studied in these data sets.

**Fig. 1:**
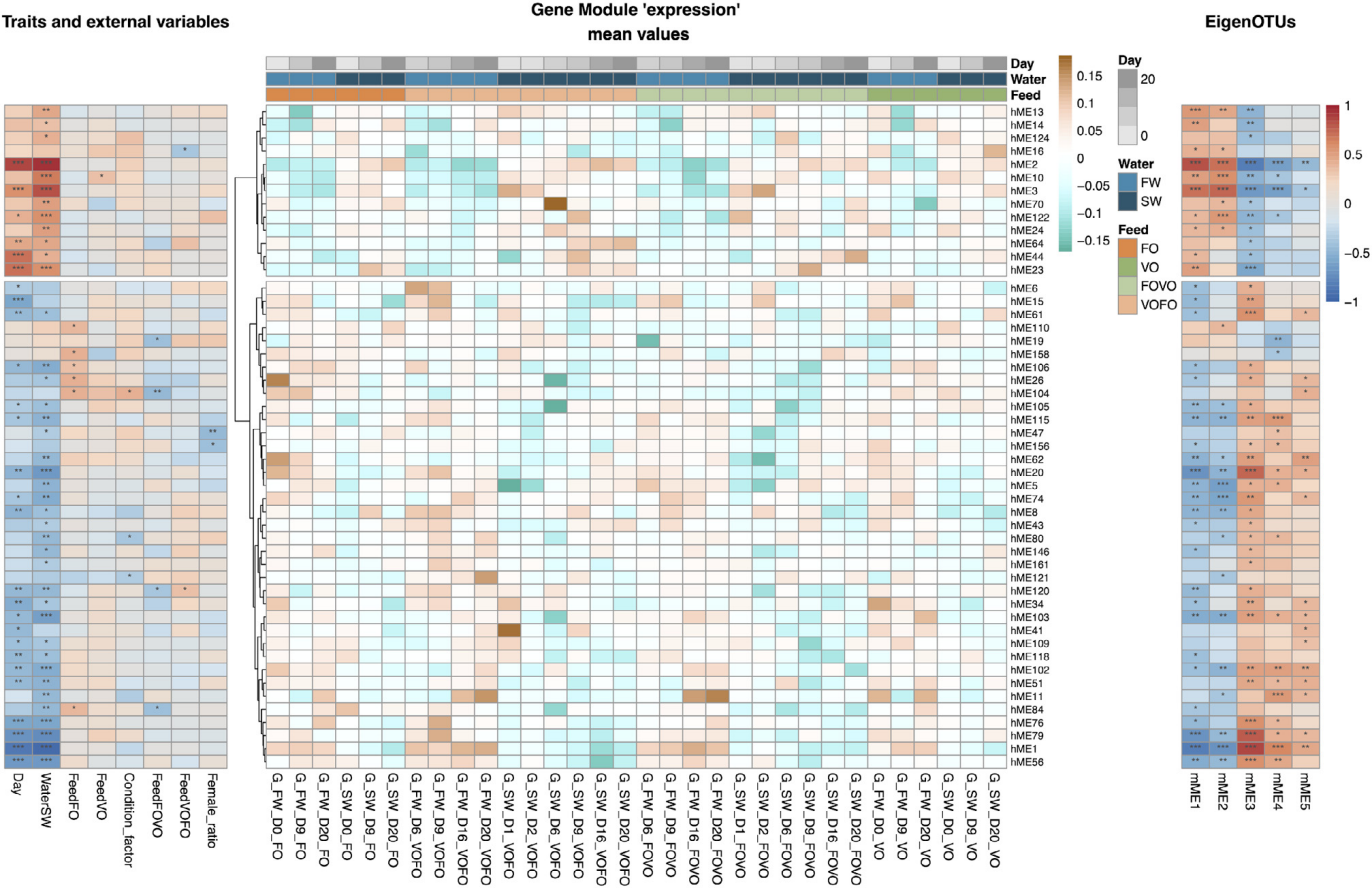
Integrated heatmap. The figure shows correlations between the host gene expression module eigengenes (hME) and the microbiome abundance module eigenOTUs (mME), as well as between hME and host traits and external variables. The central heatmap shows the ‘expression’ of the hMEs, where rows are hMEs and columns are sample groups (i.e. mean of replicates). Sample groups are ordered by feed type, then water type and finally by day. The right heatmap shows correlation strengths between hMEs and mME, while the left heatmap shows correlation strengths between hMEs and host traits/external variables. Correlations are computed across sample groups. A strong positive correlation is represented by deep red, and a strong negative correlation by deep blue. A correlation of 0 is white. Statistical significance of the correlations as indicated with stars: * p <= 0.05, ** p <= 0.01, *** p <= 0.001. (For interpretation of the references to colour in this figure legend, the reader is referred to the web version of this article.)

We developed a visualization scheme where gene module expression, transkingdom putative interactions and omics correlations to host traits and external variables are assembled into an integrated Heatmap Analysis of Holobiont Interactions (iHAHI, Fig. 1). This analysis clearly shows that the fresh-/salt-water transition explains much of the structure in both the host and microbial network. The 50 host modules with a significant association to both external variables and microbiota modules (p < 0.05) are broadly divided into two groups: group 1 contained modules of genes that to some degrees have higher expression in saltwater (i.e. positively correlated to WaterSW – Salt Water, and to Day, which is inherently linked to water type), whereas group 2 primarily have higher expression in freshwater (i.e. negatively correlated to WaterSW). Notably, the two largest host modules (represented by host Module Eigengene 1 – hME1 – and 2 – hME2) have the strongest negative/positive association to saltwater, respectively. Some of the strongest associations between host and microbial modules are also observed for these two modules. This is explained by the fact that microbial modules also are heavily influenced by the fresh-/salt-water transition: microbial modules 1 and 2 (microbial Module EigenOTU 1 – mME1 – and 2 – mME2) contain OTUs abundant in saltwater, while modules 3, 4 and 5 contains genes abundant in fresh water (Supplementary Fig. 4).

Since many host and microbiota modules correlate with the fresh-/salt-water transition, it is difficult to distinguish actual host-microbial interactions in these modules from scenarios where both host and microbial modules are independently explained by water-type. In order to gain further insight into this, we investigated gene function enrichment and OTU content in host-microbiota module-pairs abundant in saltwater (hME2 and hME3 and their correlation with mME1 and mME2) and in freshwater (hME1, hME56, hME76 and hME79 and their correlation with mME 3 and mME4).

#### 2.2.1 Host-microbiota modules abundant in saltwater

Host module 2 was highly enriched for processes such as intracellular protein transport (p = 1e-30), lipid synthesis processes such as phosphatidylethanolamine biosynthetic process (p = 2.2e-12), cholesterol biosynthetic process (p = 3.2e-10), triacylglycerol biosynthesis process (p = 3.2e-10), long-chain fatty-acyl-CoA biosynthetic process (p = 5.9e-8) and regulation of lipid storage (2e-7) while host module 3 was also enriched for lipid related processes such as triglyceride homeostasis (p = 3.2e-4), lipid oxidation (5.3e-4) and lipid catabolic process (1.1e-3) (Supplementary table 3). These processes are in line with the function of gut epithelial cells given high availability of LC-PUFAs. Microbial module 1 contains a majority of Firmicutes (37 of 45), which generally dominates the gut in salt water, but also contains Proteobacteria (4), Actinobacteria (3) and Cyanobacteria (1) (Supplementary Fig. 5). Many OTUs in this module persist across the freshwater-saltwater transition, although more highly abundant in salt- than in fresh-water, and includes as highly central nodes all four OTUs constituting the “stable core gut microbiota” previously discussed by [35] (Supplementary Fig. 5). Microbial module 2 has a different OTU profile, containing Bacteroidetes (6 of 22), Proteobacteria (6) Fusobacteria (4), Cyanobacteria (3) and Firmicutes (3). Deeper taxonomic assignments at the genus level identified many taxa associated with fresh and marine water environments (i.e. Planktothrix, Aeromonas etc), but also indicated a prevalence of Bacteroides-like populations, which are widely recognized in gastrointestinal ecosystems for their fermentative capabilities of host and plant-derived glycans [28] (Supplementary Table 2).

#### 2.2.2 Host-microbiota modules abundant in freshwater

Host module 1 is highly enriched in extracellular matrix disassembly (p < 5.6e-27) including collagen catabolic process (p < 6.4e-19). Microbial module 3 contains Proteobacteria (7), which generally dominates the gut in fresh water, but also Actinobacteria (3), Firmicutes (2), Cyanobacteria (1) and the only Synergistetes in the data set. Microbial module 4 is also dominated by Proteobacteria (7 of 8) and contains one Bacteroidetes. Both microbial modules were dominated by taxa frequently observed in water-bourne habitats with only limited examples of host-associated microbiota (i.e. Enterococcus).

Taken together, these results indicate that the environment is the primary driver of selection on gut microbiota with large numbers of genes and OTUs differentially abundant between fresh- and salt-water. Still, significant host-microbial associations related to feed, sex and growth does exist, suggesting that diet or host metabolism may play some role in shaping the co-occurrence of associated host-microbiota modules.

### 2.3 Host-microbial associations related to feed, sex and growth exists

Beyond the large effect that the fresh/salt water-transition had on both host gene expression and gut microbiota, we also identified significant associations between host gene expression and microbial communities that are linked to feed types (Feed), sex (Female_ratio) and fish weight and length (Condition_factor, see Methods) (Fig. 1). Compared to the large effects of the fresh/salt water transition, these correlations were much weaker, involved small host modules, and generally did not co-vary with water type. Nevertheless, many biological meaningful connections are evident. For example, host module hM47 is associated with the sex of the fish and contains a sprouty-related 2C gene known to have sex-biased gene expression in other fish [38].

All four feed categories (FO, VO, FO → VO, and VO → FO) had at least one significant correlation to a host-module and to a microbial module (Fig. 1). These host modules consisted of genes linked to functions such as ion transport (hM10, hM104), fatty acid binding (hM10), apoptosis (hM120), and muscle function (hM10), but also various genes associated with anti-inflammatory pathways (Supplementary table 1). For example, the switch of diet from FO to VO was particularly strongly associated with expression of hM104, which contains the ankyrin-3 gene (*ank3*) that alters Na-K-ATPase activity during chronic intestinal inflammation [37]. In line with this, increased levels of n-3 LC-PUFA (DHA and EPA) in the feed have been shown to activate anti-inflammatory processes in the gut in both mammals and fish [29], [8].

The switch of diet from VO to FO was associated with hME16 which contains interferons regulatory factor genes (*irf-3* and *irf-7*), signal transducer and activator of transcription 1 gene (*stat-1*) and interferon-induced very large GTPase 1 gene (*gvinp-1*) involved in innate and adaptive immune responses. The interferon signalling involves *irf-3* and *irf-7* which regulates the transcription of type 1 interferons (T1ifn) and leads to phosphorylation of Stat-1 and Stat-2 [14], [26]. The Stat-1 and -2 dimerze and form a complex, which enters the nuclears and affects transcription of interferon-stimulated genes (ISGs). The genes of hME16 were negatively correlated to VOFO, supporting a previous study that dietary inclusion of LC-PUFA decreases stimulated-interferon production [17]. An alternation of T1IFN signalling in host could change the composition of commensal microorganisms in the host intestine, and conversely the intestinal microbial community also plays an important role for maintaining a stable T1IFN production at mucosal surfaces [14]. Furthermore, a study in mice also showed that the colonisation of microbiota is important for the development of T1IFN signalling systems at early stages [13].

Host module 16, associated with VOFO and two microbial modules (mME1 and mME2), contains a butyrate response factor 1 gene (NCBI locus identifier 100195422). This gene is known to be regulated by changes in butyrate levels in colorectal cells in mammals [27]. Interestingly, within microbial module 1 we identified an OTU affiliated to Faecalibacterium (OTU_45), a renowned butyrate-producing beneficial bacterium in the mammalian gut that is commonly associated with gastrointestinal health [12]. Closer inspection of OTU45 and butyrate response factor 1 demonstrated a stronger correlation between these than between the modules themselves (p = 0.001). The predicted hM16-mME1 interaction hence represents an interesting candidate for a functional link between microbial metabolism and host-gene regulation.

### 2.4 Host-microbial associations after removing large effects

Because the fresh-to-salt water transition in the dataset has an overwhelmingly large effect on gene expression and microbial composition, it is plausible that other associations have remained undiscovered in the initial analysis described above. To gain further insight into host-microbiota associations, we therefore reran our pipeline on omics data subjected to a method designed to remove large effects (see Methods). Interestingly, this revealed a large number of small modules for both the host (123 host modules, Supplementary figures 6) and microbiota networks (89 microbiota modules, Supplementary figure 7). These modules included a large proportion of genes (586 of 1492 genes, Supplementary table 4) and OTUs (156 of 228 OTUs, Supplementary table 5) not assigned to modules in the initial analysis, and thus represented a potential for new discoveries.

**Fig. 2:**
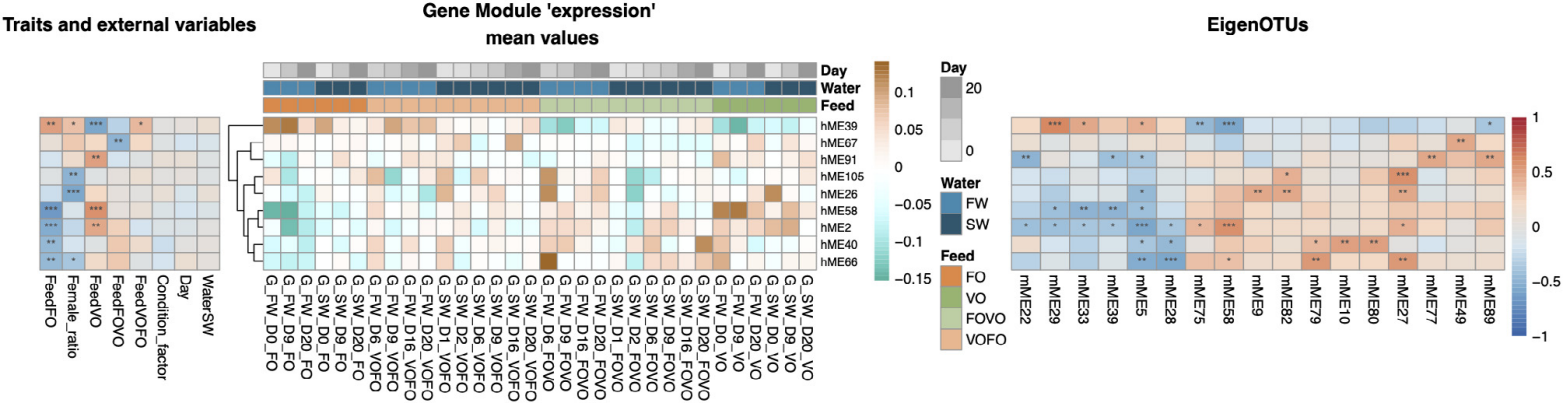
Integrated heatmap with large effects removed. The figure shows correlations between the host gene expression module eigengenes (hME) and the microbiome abundance module eigenOTUs (mME), as well as between hME and host traits and external variables. The central heatmap shows the ‘expression’ of the hMEs, where rows are hMEs and columns are sample groups (i.e. mean of replicates). Sample groups are ordered by feed type, then water type and finally by day. The right heatmap shows correlation strengths between hMEs and mME, while the left heatmap shows correlation strengths between hMEs and host traits/external variables. Correlations are computed across sample groups. A strong positive correlation is represented by deep red, and a strong negative correlation by deep blue. A correlation of 0 is white. Statistical significance of the correlations as indicated with stars: * p <= 0.05, ** p <= 0.01, *** p <= 0.001. (For interpretation of the references to colour in this figure legend, the reader is referred to the web version of this article.)

Fig. 2 clearly shows that removing large effects deemphasized association related to water type and sampling day, and instead revealed several strong associations (p < 0.01) involving nine host modules, 17 microbiota modules and host variables linked to feed and sex. Here we focus on modules with associations to both VO and FO feeds: Host module 2 is enriched for genes with a role in the cholesterol biosynthetic process (p = 1E-30, Supplementary table 6), which is the same enrichment as for genes highly expressed in salt water in the initial analysis (host module 2 in Fig. 1). However, unlike in the initial analysis, host module 2 after removing large effects (Fig. 2) has a strong association to feed, with a positive correlation to VO feed and a negative correlation to FO feed. The same feed association can be found for host module 58. A higher requirement for endogenous cholesterol has previously been suggested to be an effect of dietary VO which is naturally devoid of cholesterol [21], [15]. Further supporting this, host module 58 includes a scavenger receptor class B type I gene (sr-bi) that suggest a higher requirement of cholesterol absorption in the intestine in VO [6], [3]. Furthermore, host module 39, displaying the reverse association to feed (negative correlation to VO feed and positive to FO feed), is enriched for positive regulation of epithelial to mesenchymal transition (p = 0.00016). Host module 39 includes two genes, beta,beta-carotene 9′,10′-oxygenase-like (*bco2*) involved in cleavage of carotenoids and lecithin retinol acyltransferase-like (*lrat*) involved in esterification of carotenoids. The higher expression of genes involved in carotenoid metabolism could be linked to LC-PUFA levels in FO. A previous study has shown that sterol esters containing LC-PUFA have higher bioavailability than vegetable oil fatty acid esters [23]. Multiple microbial taxa were also observed to associate with host modules 2, 58 and 39, including several lineages affiliated to gastrointestinal commensals such as *Paraeggerthella* (OTU_230, mME5) [25] and those linked to fermentation and/or fatty acid utilization (e.g. *Tepidimicrobium:* OTU_311, mME5 and OTU_257, mME58) [40]. However, it must be noted that little phenotypic and genomic information exists for these genera in any environment notwithstanding the salmon gut for which there exists no data. This current lack of data for microbial function makes it problematic to infer definitive predictions of metabolic functions based on our 16S rRNA data and to connect their putative associations to host modules. It is hoped that these knowledge gaps will quickly be filled as improvements to microbiome genome inventories are occuring at a rapid pace.

Taken together, we successfully removed the dominating effect of water type and sampling day from the host and microbiota omics data, and then showed that our network-based framework could reveal hitherto undiscovered host-microbiota-feed associations in this data.

## 3 Discussion

Previous studies have shown that both changes in feed composition and migration between fresh- and sea-water environments remodel salmon gut tissue function as well as gut microbial composition [15], [21], [22], [41] Yet, during these significant events there is little to no knowledge about potential interconnections between the microbial community functions and salmon gene regulation and gut physiology. To close this knowledge gap there is a pressing need to co-analyze host and microbiome responses in a unified manner.

Here we used matching host gene expression and microbial level measurements to search for host-microbiota interactions using a novel computational method combining network-based dimensionality reduction for analysing holobiont omics data and an integrated Heatmap Analysis of Holobiont Interactions (iHAHI) for visualizing putative interactions. Although this method was applied herein to a salmon dataset, it can be applied to any holo-omic dataset in animals or plants. In fact, the R code is available as modules (R Markdown files) each performing one step in the computational analysis pipeline (Supplementary Fig. 2) including generating all figures presented as part of this article. Using this pipeline, we find that it is difficult to separate salmon host-microbiota interactions from independent effects in the host and the microbiota both caused by the fresh-/salt-water transition. Although in seawater, we observed OTUs affiliated to the *Bacteroides*, which are well recognized for their commensal activity within gastrointestinal ecosystems. In the context of dietary changes, previous traditional single-omics analysis showed that while host gene regulation plays a role in adopting salmon to different LC-PUFA availability, the gut microbiota composition seems not to play a major role. In this study we similarly observed correlations between host gene expression and feed, however by introducing an additional layer of data encapsulating gut microbiome composition, we revealed putative interactions that arise from possible microbiome butyrate-producing activity. Collectively, our data therefore suggests that perhaps overlooked dietary-linked commensal populations that are critical to mammalian nutrition and wellbeing (such as *Bacteroides* and *Faecalibacterium*) are also important within salmon. Finally, we show that when large effects are removed from our analysis we increase the resolution of network associations, revealing previously hidden and possibly important biological interactions.

Our method for detecting robust interactions between host and microbiota was here used to find correlated modules of host genes and microbiota OTUs. While individual host genes and microbiota OTUs might still exhibit signals of interactions, the use of microbial taxa as a marker as opposed to microbial gene function may well mask many critical functional interactions that exist between metabolic pathways that have not yet been attributed to taxonomically-described salmon gut microbiota. In addition, the presence of an OTU or gene/pathway does not automatically infer activity. The current lack of data for microbial function makes it problematic to infer definitive predictions of metabolic functions based on 16S rRNA data and to connect their putative associations to host modules. It is hoped that these knowledge gaps will quickly be filled as improvements to microbiome genome inventories are occuring at a rapid pace. Irrespective, we argue that our method easily can encompass host and microbial gene expression data once representative datasets become available for salmon.

## 4 Materials & methods

### 4.1 Materials

The omics data were generated using 367 samples from a long term feeding trial of farmed Atlantic salmon (*Salmo Salar*) (for experimental design see Supplementary Fig. 1). The salmon were raised on two contrasting diets: one vegetable oil diet (VO) with low amounts of long chain polyunsaturated fatty acids (LC-PUFA), containing a 1.8:1 ratio of linseed oil and palm oil, and one fish oil diet (FO) high in LC-PUFA, based on fish oil from the North Atlantic [15], [35]. The fish were switched to the contrasting diet (VO to FO and *vice versa*) at ~ 50 g in freshwater, and tissue samples were taken 1, 2, 5, 9, 16 and 20 days after the diet switch (Supplementary Fig. 1b). The control fish on the original diets was put through the smoltification process and then transferred to seawater. Another diet switch trial was performed in seawater when the fish reached ~ 200 g, and tissue samples were again taken at 1, 2, 6, 9, 16 and 20 days after the diet switch. Samples of the control tanks were taken at 0, 5, 9 and 20 days after diet switch in freshwater and at 0, 9 and 20 days in seawater.

Atlantic salmon RNA-seq reads from the gut epithelial tissue samples were taken from [15] and are available in the European Nucleotide Archive (ENA) as project PRJEB24480. Transcript expression values were quantified using Salmon [32]. Corresponding gut microbiota 16S rRNA amplicon sequencing reads were taken from [35] and are available in the Sequence Read Archive (SRA) under accession number SRP119730. OTUs were identified from 16S sequences using the USEARCH pipeline with 97% sequence identity. Classifications were done with the R-package *microclass*.

### 4.2 Methods

The developed computational analysis pipeline is visualized in detail in Supplementary Fig. 2.

#### 4.2.1 Data preprocessing

Transcript expression counts were summed for each gene. Expression values were normalized across samples using the Trimmed mean of M values (TMM)-method [33], and log2-transformed. Genes with expression levels below 1.0 in all samples and/or with a standard deviation less than 0.15 were removed before network inference. This reduced the number of genes from 48,057 to 37,408.

We retained OTUs that contribute at least 0.005% of the total microbial abundance. This reduced the number of OTUs from 1152 to 296 and the number of zeros in the abundance table by 50%. OTU abundances was normalized using the Cumulative Sum Scaling (CSS) method from the Bioconductor package *metagenomeSeq,* which by default log2-transforms the data.

Samples were first normalized with a min-max normalization (Cao, Stojkovic, and Obradovic 2016) and then clustered with average distance to identify outliers. Two samples were removed: RNA-seq sample G_FW_D20_FOVO_2 and 16S sample G_FW_D9_FOVO_2.

#### 4.2.2 Network inference

For network inference, we used the Weighted Gene Co-expression Network Analysis (WGCNA) R package [24] and the function *blockwiseModules* with the bicor correlation measure and parameters maxBlockSize = 10000, networkType = “signed”, TOMType = “signed”, corType = “bicor”, maxPOutliers = 0.05, replaceMissingAdjacencies = TRUE, pamStage = F, deepSplit = 4, minModuleSize = 2, minKMEtoStay = 0.5, minCoreKME = 0.5, minCoreKMESize = 2, reassignThreshold = 0 and mergeCutHeight = 0.4/0.5 (for microbiota/host respectively).

Our analysis relies heavily on network modules, and hence the parameters related to module detection and trimming influence the results. Briefly, deepSplit controls the sensitivity of the module detection approach by hierarchical clustering, with a value of 1 being the least sensitive and 4 being the most sensitive. minModuleSize controls the minimum size of modules in the clustering step. Nodes with a correlation to the module eigennode (KME) lower than minKMEtoStay are trimmed from the module, and the module is deleted if it does not have a core of at least minCoreKMESize nodes (with core nodes being defined as having a KME greater than minCoreKME). Finally, different modules with eigennodes that correlate above the 1 – mergeCutHeight threshold are merged. Note that the final modules can be smaller than minModuleSize due to trimming (but not smaller than minCoreKMESize), and that they can include nodes with a KME lower than minKMEtoStay due to module merging. Our parameters were set to detect highly correlated and potentially small modules initially, thus not missing interesting profiles displayed by few genes/OTUs, and then to apply an aggressive merging threshold to avoid dealing with highly redundant modules in downstream analysis.

Network centrality of genes and OTUs were calculated using the function *intramodularConnectivity.fromExpr*.

The removal of large-effect variables using the PC-correction method available in the R-package *sva* [31], which is a method developed for co-expression networks, is implemented in the code. The number of latent variables to be removed is estimated using the permutation-based approach implemented in the *num.sv* function and the variables can then be regressed out using the function *sva_network*.

#### 4.2.3 Host variables

We correlated a set of host variables/traits with the omics data including day, feed, water type, sex, weight and length. The categorical feed-variable has four values: vegetable oil (VO), fish feed (FO) and the two transitions from VO to FO (VOFO) and from FO to VO (FOVO). To allow correlation analysis we expanded this variable into four new binary variables using “one hot encoding” with each new variable taking values 1 (e.g. VO) and 0 (e.g. not VO). Instead of using host weight and length directly, we calculated the condition factor (CF) of the fish, which is considered an indirect measure of fish fatness [5], [2]. The new variable CF is a continuous variable:

$$\mathrm{CF}=\frac{10^{N}\cdot W}{L^{3}}$$

where W is the weight of the fish in grams, L is the length of the fish in millimeters and N is a constant used to bring the range of output values close to 1. The value of N differs from N = 2 [2] to N = 5 [5]. Here we used a value of N = 4 which gives the expected range.

#### 4.2.4 Integrated heatmap analysis of holobiont interactions

We devised and implemented a heatmap analysis to visualize and predict putative holobiont interactions by integrating host and microbiota module eigennodes with host variables (see Fig. 1, Fig. 2). To obtain reliable estimates of the significance of associations between host eigengenes and both host variables and eigenOTUs, correlations were computed after averaging the expression/abundance values of replicates. Correlations and p-values were computed using the R function *cor.test* using the Spearman correlation for the ordinal day-variable and the default Pearson correlation for all other variables.

#### 4.2.5 Gene ontology enrichment

The R package TopGO [1] was used to test for gene function enrichment.

#### 4.2.6 Code availability

The R-markdown files and R-scripts are available at: https://gitlab.com/M.strand/wgcna_host_microbiome.

## CRediT authorship contribution statement

**Marius A. Strand:** Methodology, Software, Formal analysis, Data curation, Writing - original draft, Visualization. **Yang Jin:** Investigation, Writing - review & editing. **Simen R. Sandve:** Conceptualization, Investigation, Writing - review & editing, Supervision, Funding acquisition. **Phil B. Pope:** Conceptualization, Investigation, Writing - review & editing, Supervision, Funding acquisition. **Torgeir R. Hvidsten:** Conceptualization, Validation, Writing - original draft, Supervision.
