# Supplementary material for: Transkingdom network analysis provides insight into host-microbiome interactions in Atlantic salmon
Source: Comput Struct Biotechnol J. 2021 Jan 29;19:1028–34. doi: 10.1016/j.csbj.2021.01.038 (PMC7876536; doi:10.1016/j.csbj.2021.01.038)
Supplement: Supplementary data 1 [file mmc1.pdf]

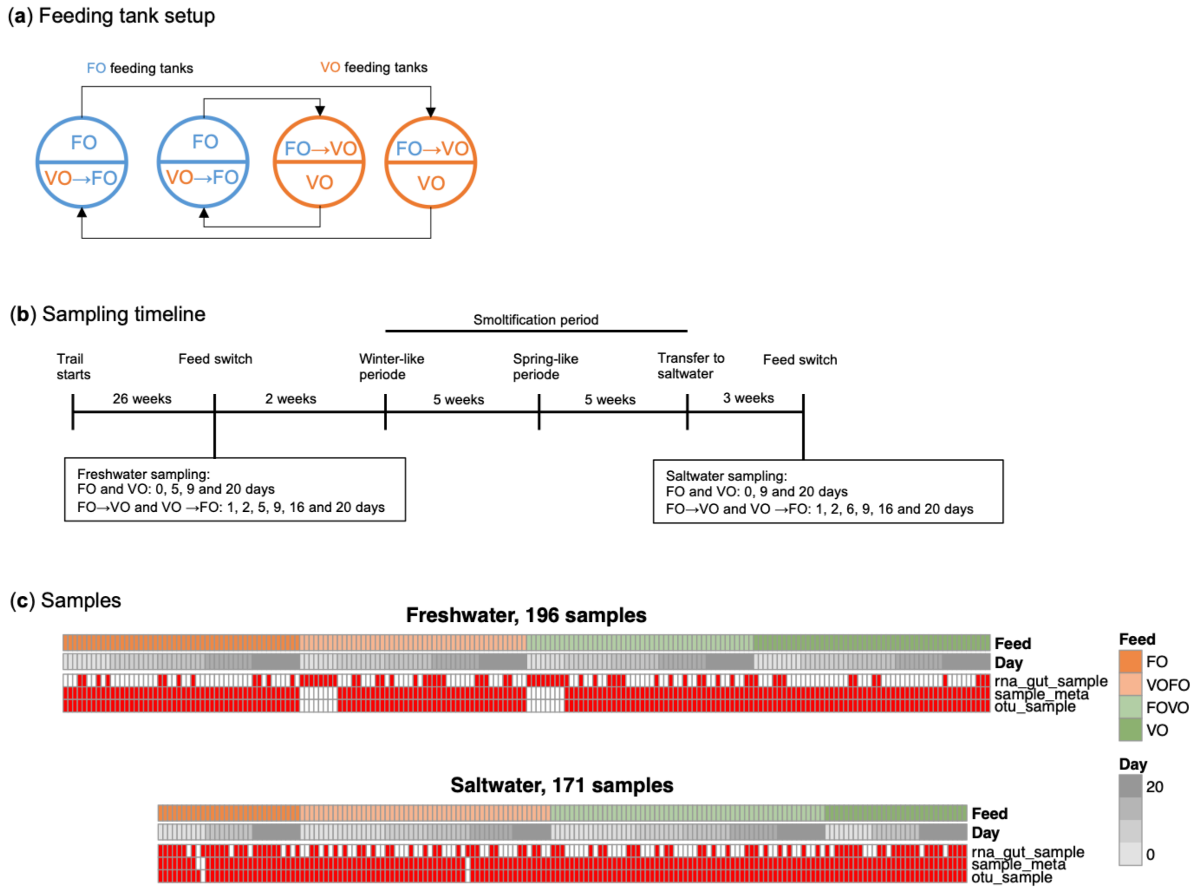

Supplemental figure 1: Overview of experimental set up and timeline. *(a) Atlantic salmon fry was reared in four different tanks which each had two compartments. Fish in two tank halves were continuously given feed with vegetable oil as its lipid source (VO), fish in the other two tank halves were given feed with fish oil (FO). Switching feed involved taking fish from VO and FO tanks and moving them to empty tank haves with the opposite feed regimen. Only fish from the non-switched tanks were transferred to new tanks containing saltwater after smoltification where the same process was repeated. (b) Experiment and sampling timeline. In total the experiment lasted ~47 weeks. There were two sampling times each lasting 20 days, one in freshwater 26 weeks after hatching and one three weeks after introduction to saltwater. (a) and (b) are adapted from Gillard et al. (2018). c) Sample overview. VO: Vegetable oil, low LC-PUFA. FO: Fish oil, high LC-PUFA. Red indicates that there is a sample, white indicates that there is not.*

General Schematic

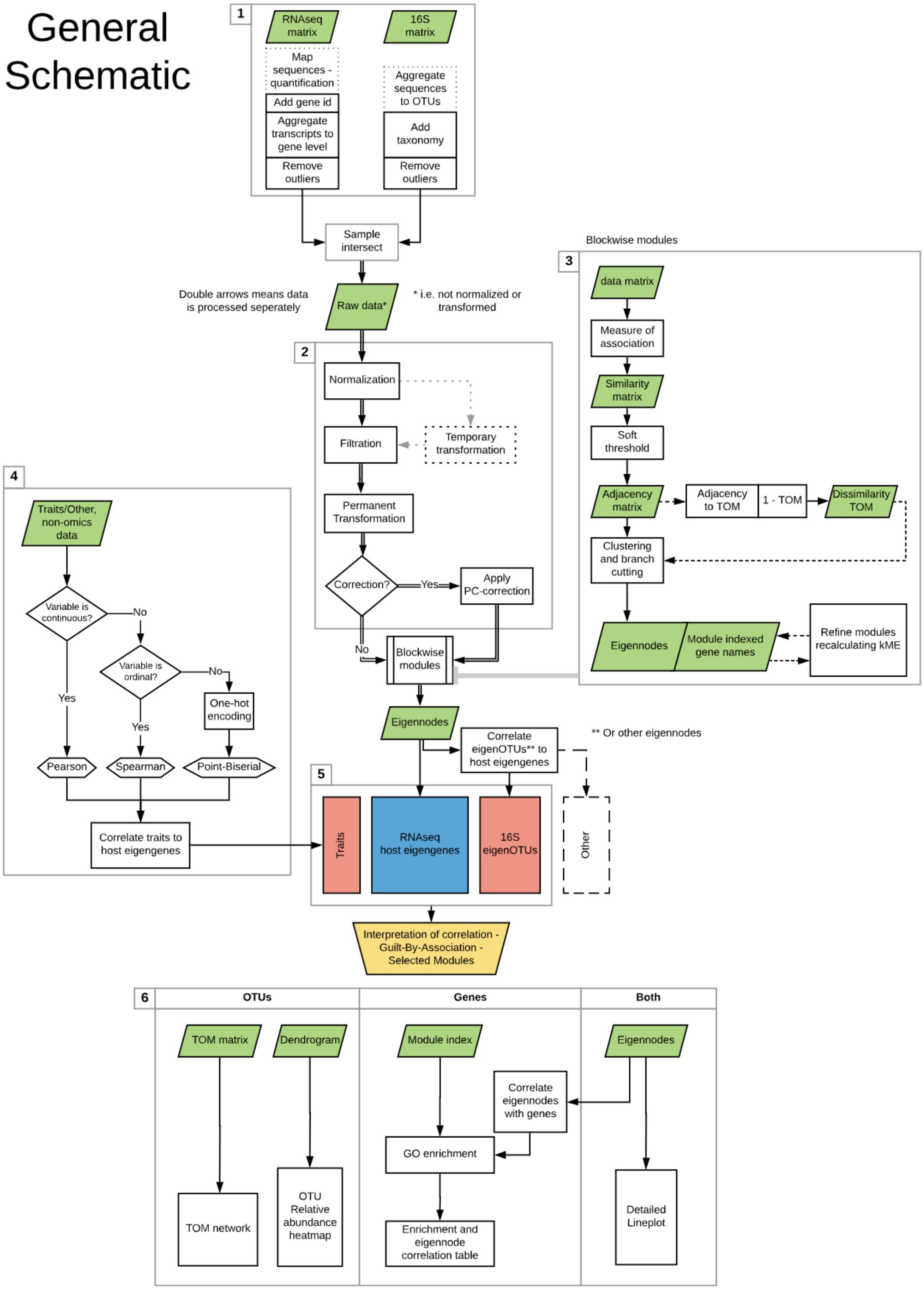

Supplementary figure 2: General pipeline for integrating host-microbiota omics data. *Numbers indicate the progression of the pipeline. 1. Input and preparation of data, 2. Normalization and transformation, 3. Construction of weighted networks and identification of modules, 4. Processing of external variables, 5. Visualization of correlations between modules and external variables, 6. Detailed exploration.*

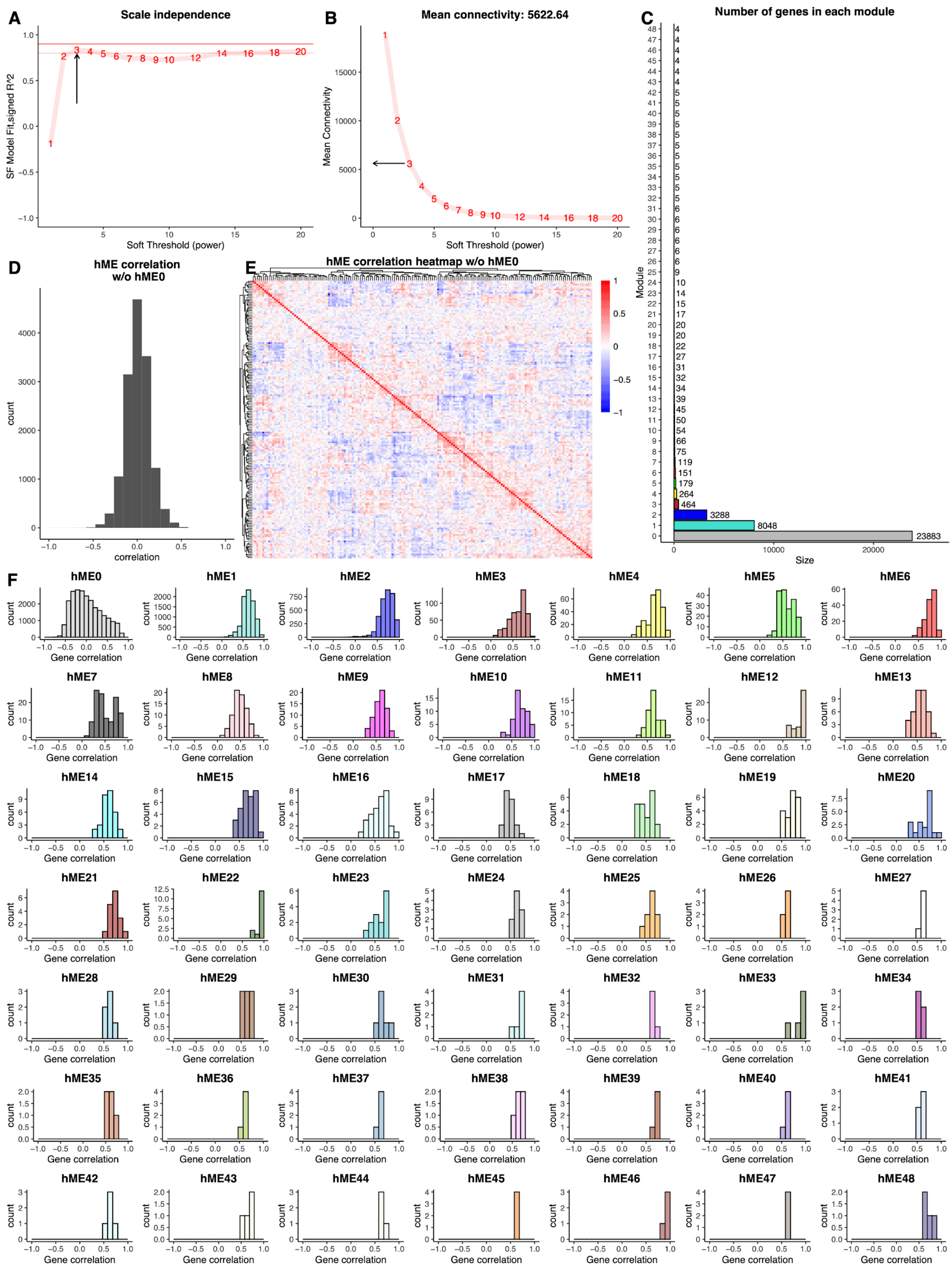

A
Scale independence
SF Model Fit,signed R^2
Soft Threshold (power)
B
Mean connectivity: 5622.64
Mean Connectivity
Soft Threshold (power)
C
Number of genes in each module
Module
Size
D
hME correlation
w/o hME0
count
correlation
E
hME correlation heatmap w/o hME0
F
hME0
hME1
hME2
hME3
hME4
hME5
hME6
hME7
hME8
hME9
hME10
hME11
hME12
hME13
hME14
hME15
hME16
hME17
hME18
hME19
hME20
hME21
hME22
hME23
hME24
hME25
hME26
hME27
hME28
hME29
hME30
hME31
hME32
hME33
hME34
hME35
hME36
hME37
hME38
hME39
hME40
hME41
hME42
hME43
hME44
hME45
hME46
hME47
hME48
Gene correlation

Supplementary figure 3: Diagnostic plots for host gene co-expression network. A) *Scale independence. The plot shows how the degree distribution of networks at different soft-thresholding powers (βs, betas) fits a scale-free distribution (R^2). The arrow shows the beta value chosen for the network.* ***B)*** *Mean connectivity. The plot shows the mean connectivity of the networks as a function of soft-thresholding powers. The chosen power is marked with an arrow, and the numerical value of the mean connectivity is shown in the title of the plot.* ***C)*** *Module size. The plot visualizes the size of the 49 largest modules i.e. how many genes each module contains. "Module 0" contains genes that were not assigned to any well-defined module.* ***D)*** *Module similarity. A histogram of the correlations between host module eigengenes (hMEs, "module 0" is left out).* ***E)*** *Module similarity. A heatmap visualizing the pairwise correlations between module eigengenes.* ***F)*** *Module homogeneity**.** Histograms of correlations between each of the 49 largest module's eigengene and the genes in the module.*

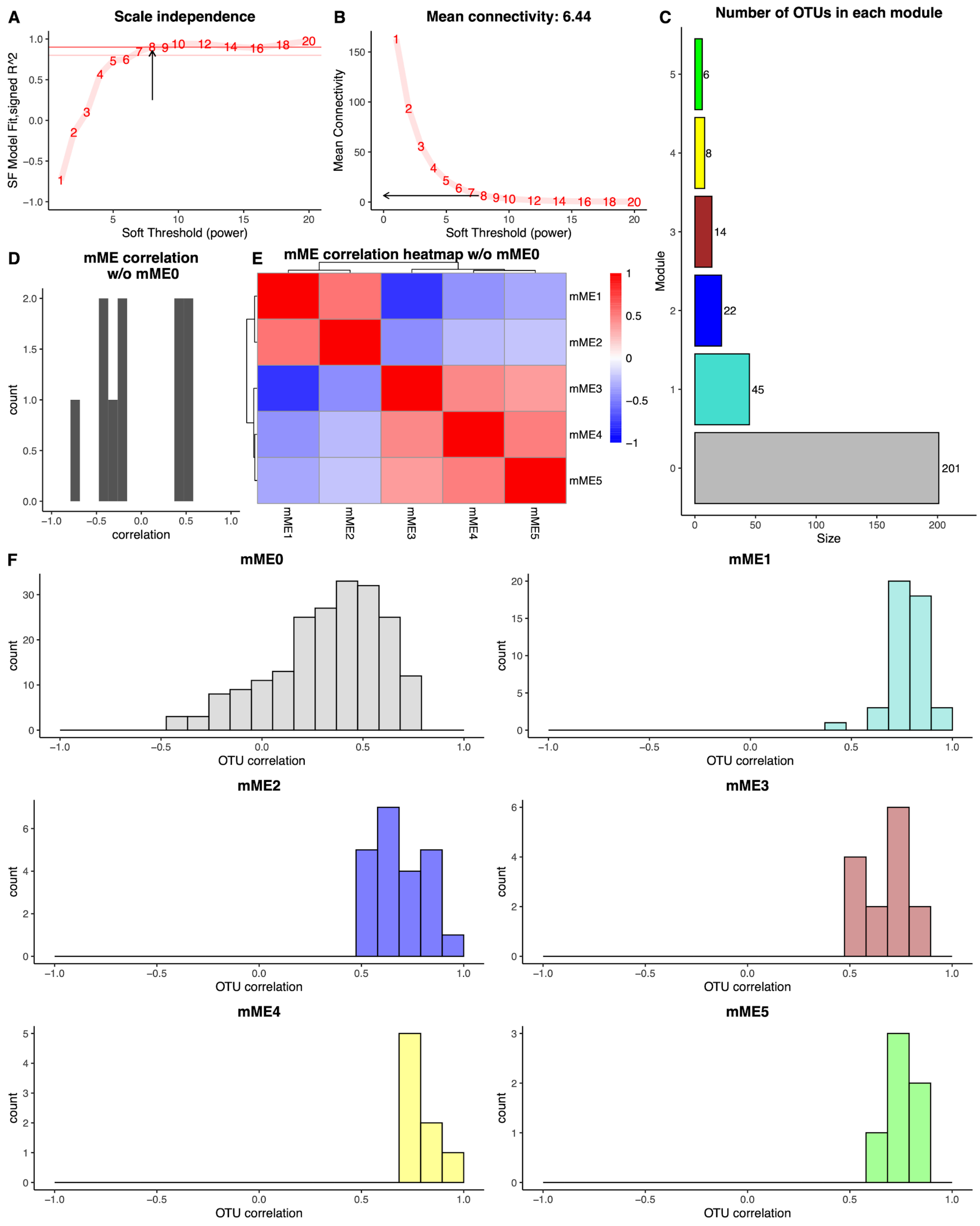

Supplementary figure 4: Diagnostic plots for gut microbiome co-abundance network. A) *Scale independence. The plot shows how the degree distribution of networks at different soft-thresholding powers (βs, betas) fits a scale-free distribution (R^2). The arrow shows the beta value chosen for the network.* ***B)***

Mean connectivity. The plot shows the mean connectivity of the networks as a function of soft-thresholding powers. The chosen power is marked with an arrow, and the numerical value of the mean connectivity is shown in the title of the plot. ***C)*** *Module size. The plot visualizes the size of the modules i.e. how many OTUs each module contains. "Module 0" contains OTUs that were not assigned to any well-defined module.* ***D)*** *Module similarity. A histogram of the correlations between host module eigenOTUs (mMEs, "module 0" is left out).* ***E)*** *Module similarity. A heatmap visualizing the pairwise correlations between module eigenOTUs.* ***F)*** *Module homogeneity**.** Histograms of correlations between each module's eigenOTU and the OTUs in the module.*

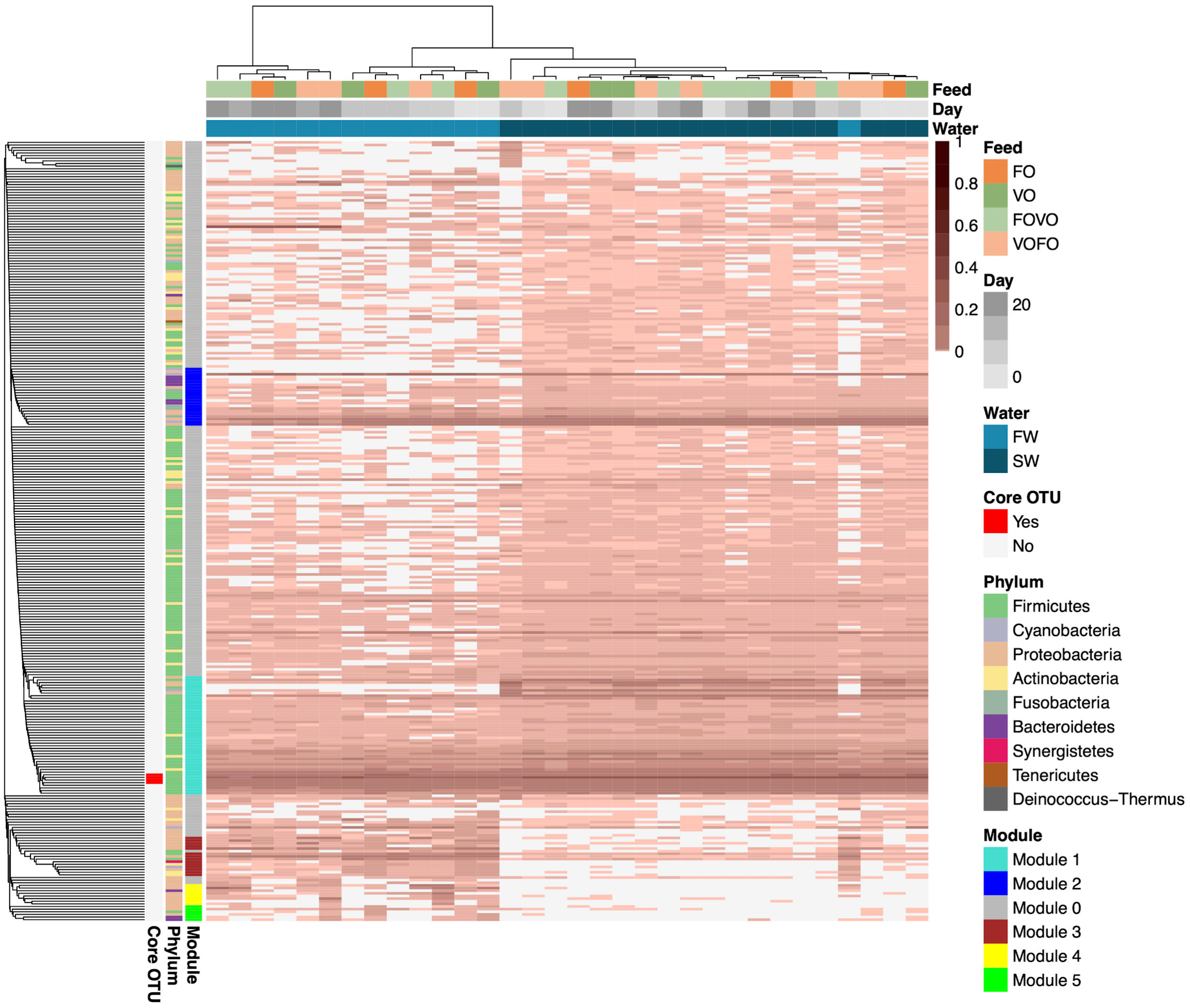

Supplementary figure 5: A heatmap of OTU relative abundances. *OTUs are rows and samples are columns (mean values of replicates). The color scheme has been adjusted so that zero abundance is light grey, while any abundance >0 is colored with intervals which emphasises the low end of the abundance. There are three row-annotation-bars: The first shows in red the four core OTUs found in Rudi et al. (2018), the second shows the determined phyla of the OTU, the third shows module membership.*

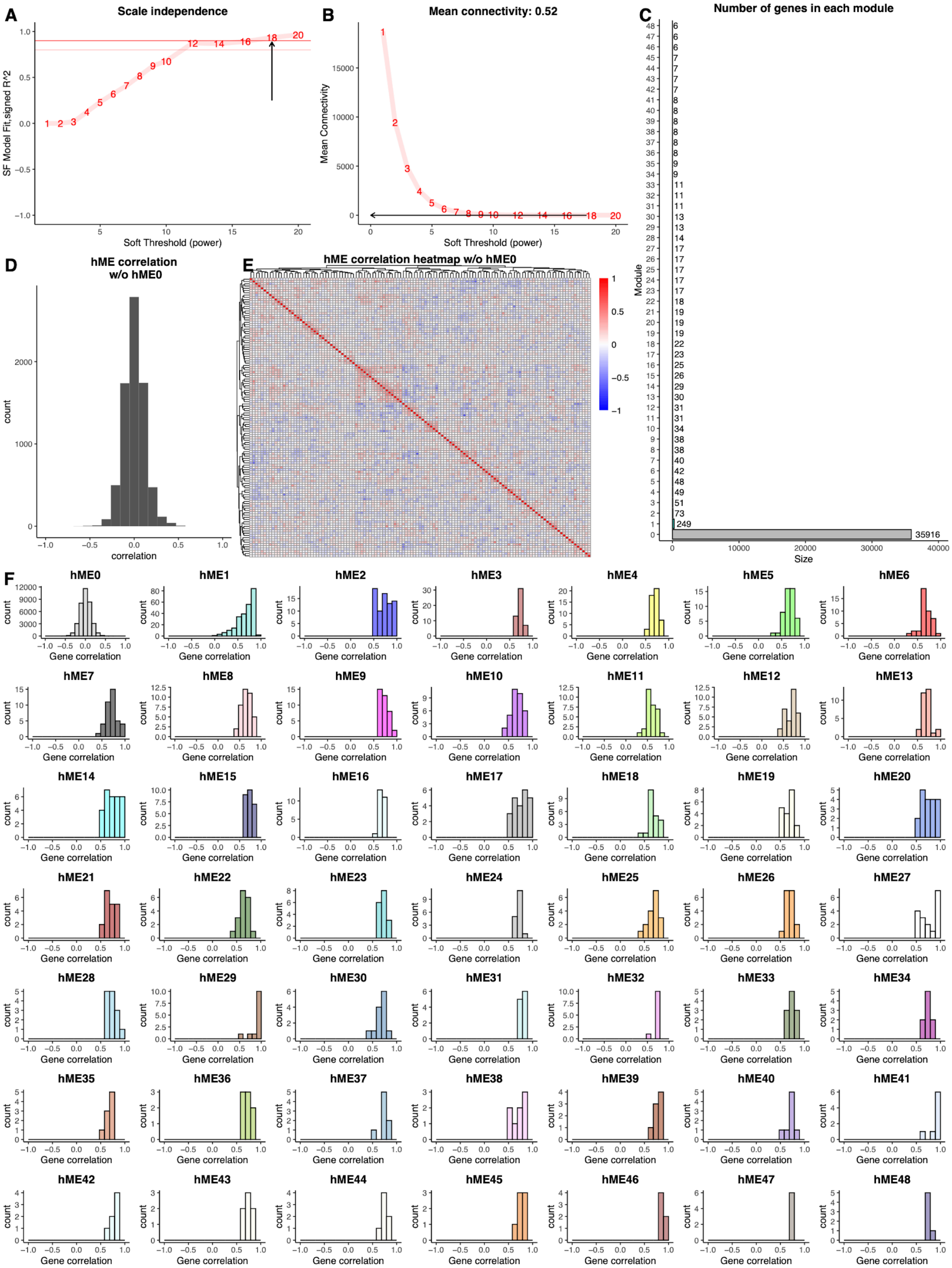
A
Scale independence
SF Model Fit,signed R^2
Soft Threshold (power)
B
Mean connectivity: 0.52
Mean Connectivity
Soft Threshold (power)
C
Number of genes in each module
Module
Size
35916
249
D
hME correlation
w/o hME0
count
correlation
E
hME correlation heatmap w/o hME0
F
hME0
hME1
hME2
hME3
hME4
hME5
hME6
hME7
hME8
hME9
hME10
hME11
hME12
hME13
hME14
hME15
hME16
hME17
hME18
hME19
hME20
hME21
hME22
hME23
hME24
hME25
hME26
hME27
hME28
hME29
hME30
hME31
hME32
hME33
hME34
hME35
hME36
hME37
hME38
hME39
hME40
hME41
hME42
hME43
hME44
hME45
hME46
hME47
hME48
Gene correlation
count

Supplementary figure 6: Diagnostic plots for host gene co-expression network after removing large effects. *See figure text of supplementary figure 3 for details.*

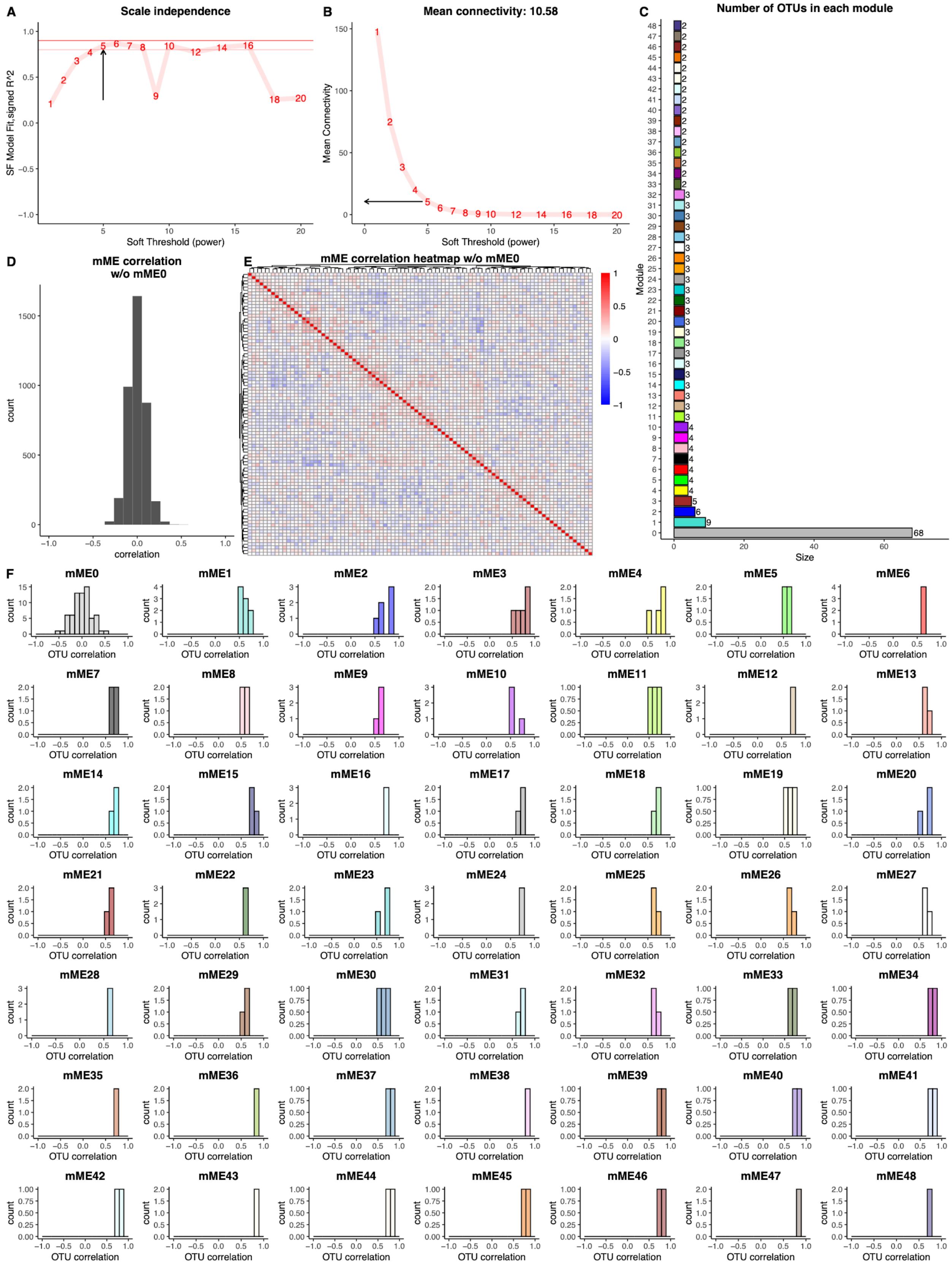
A
Scale independence
SF Model Fit,signed R^2
Soft Threshold (power)
B
Mean connectivity: 10.58
Mean Connectivity
Soft Threshold (power)
C
Number of OTUs in each module
Module
Size
D
mME correlation
w/o mME0
count
correlation
E
mME correlation heatmap w/o mME0
F
mME0
mME1
mME2
mME3
mME4
mME5
mME6
mME7
mME8
mME9
mME10
mME11
mME12
mME13
mME14
mME15
mME16
mME17
mME18
mME19
mME20
mME21
mME22
mME23
mME24
mME25
mME26
mME27
mME28
mME29
mME30
mME31
mME32
mME33
mME34
mME35
mME36
mME37
mME38
mME39
mME40
mME41
mME42
mME43
mME44
mME45
mME46
mME47
mME48
OTU correlation

Supplementary figure 7: Diagnostic plots for gut microbiome co-abundance network after removing large effects. *See figure text of supplementary figure 4 for details.*

Supplementary table 1: Host modules*. The table lists the genes in each host module. Four network centrality measures are given (kTotal: network connectivity, kWithin: intramodular network connectivity, kOut: kTotal - kWithin, kDiff: kWithin - kOut) in addition to gene id and gene description. Genes within each module are sorted by kWithin-centrality.*

Supplementary table 2: Microbiota modules*. The table lists the OTUs in each microbiota module. Four network centrality measures are given (kTotal: network connectivity, kWithin: intramodular network connectivity, kOut: kTotal - kWithin, kDiff: kWithin - kOut) in addition to OTU name and taxonomical classification (Domain, Phylum, Class, Order, Family, Genus, Species).OTUs within each module are sorted by kWithin-centrality.*

Supplementary table 3: GO enrichment of host modules. *The table shows the 50 most significantly enriched GO terms for each module. In addition to GO id and GO term, the table contains columns for GO statistics: Observed: the number of genes that are annotated with the given GO term and that have a correlation with the eigengene of the module ≥ 0.8. Expected = the expected (i.e. expected by chance) number of genes that are annotated with the given GO term and that have a correlation with the eigengene of the module ≥ 0.8. P-value: calculated with the weight01 algorithm and Fisher's exact test. Median/Max corr: the median/maximum correlation between the eigengene and genes in the GO test.*

Supplementary table 4: Host modules after removing large effects*. See table text of supplementary table 1 for details.*

Supplementary table 5: Microbiota modules after removing large effects*. See table text of supplementary table 2 for details.*

Supplementary table 6: GO enrichment of host modules after removing large effects*. See table text of supplementary table 3 for details.*
